# Association between Red Blood Cell Distribution Width and Obstructive Sleep Apnea Syndrome: A Systematic Review and Meta-Analysis

**DOI:** 10.3390/jcm12093302

**Published:** 2023-05-05

**Authors:** Biagio Di Lorenzo, Maria Carmina Pau, Elisabetta Zinellu, Arduino A. Mangoni, Panagiotis Paliogiannis, Pietro Pirina, Alessandro G. Fois, Ciriaco Carru, Angelo Zinellu

**Affiliations:** 1 Department of Biomedical Sciences, University of Sassari, 07100 Sassari, Italy; 2 Department of Medicine, Surgery and Pharmacy, University of Sassari, 07100 Sassari, Italy; 3 Clinical and Interventional Pulmonology, University Hospital of Sassari (AOU), 07100 Sassari, Italy; 4 Discipline of Clinical Pharmacology, College of Medicine and Public Health, Flinders University, Bedfor Park, SA 5042, Australia; 5 Department of Clinical Pharmacology, Flinders Medical Centre, Southern Adelaide Local Health Network, Bedford Park, SA 5042, Australia; 6 Quality Control Unit, University Hospital of Sassari (AOU), 07100 Sassari, Italy

**Keywords:** red blood cell distribution width, obstructive sleep apnea syndrome, biomarker, disease severity

## Abstract

Although polysomnography is the gold standard method to diagnose obstructive sleep apnea syndrome (OSAS), there is an ongoing quest for simpler and relatively inexpensive biomarkers of disease presence and severity. To address this issue, we conducted a systematic review of the potential diagnostic role of the red blood cell distribution width (RDW), a routine hematological parameter of red blood cell volume variability, in OSAS. A total of 1478 articles were initially identified in the databases PubMed, Web of Science, Scopus, Embase, and Google Scholar, from their inception to February 2023, and 20 were selected for final analysis. The RDW was significantly higher in OSAS than in non-OSAS subjects (SMD = 0.44, 95% CI 0.20 to 0.67, *p* < 0.001; low certainty of evidence). In univariate meta-regression, the mean oxygen saturation (SpO_2_) was significantly associated with the effect size. No significant between-group differences were observed in subgroup analyses. Notably, in OSAS subjects, the RDW SMD progressively increased with disease severity. In conclusion, these results suggest that the RDW is a promising biomarker of OSAS (PROSPERO registration number: CRD42023398047).

## 1. Introduction

Obstructive sleep apnea syndrome (OSAS) is characterized by recurrent episodes of complete (apnea) or partial (hypopnea) obstruction of the upper airways during sleep, which lead to repeated cycles of hypoxia-reoxygenation [1,2]. In OSAS, it has been reported that intermittent hypoxia is associated with systemic oxidative stress, activation of pro-inflammatory factors, endothelial dysfunction, and metabolic alterations [3]. Though the mechanisms involved are not fully understood, oxidative stress and chronic systemic inflammation in OSAS patients play a critical role in the occurrence of adverse outcomes, mostly cardiovascular morbidity [4]. In the U.S., OSAS affects approximately 26% of subjects aged between 30 and 70 years [5], although this figure is likely an underestimate [6]. 

Polysomnography (PSG), the gold standard test for the diagnosis of OSAS [7], defines OSAS severity as the number of apnoeic or hypopnoeic events per hour of sleep (apnea hypopnea index (AHI); mild: 5 ≤ AHI < 15; moderate: 15 ≤ AHI < 30; severe AHI ≥ 30) [8]. However, as PSG requires access to specific diagnostic centers and equipment, there are increasing efforts to identify new, more accessible, respiratory and/or circulating biomarkers of OSAS [9,10,11,12,13,14]. Among them, the hematological indices white blood cell count (WBC), neutrophil count, lymphocyte count, mean platelet volume (MPV), platelet distribution width (PDW), and red blood cell distribution width (RDW) have been proposed as alternative markers to those normally used clinically, e.g., interleukin-6 (IL6) and C-reactive protein, to evaluate the burden of inflammation in OSAS [15]. In particular, the RDW, a routine parameter of red blood cell (RBC) morphology that is calculated by dividing the standard deviation of RBC volumes by their mean corpuscular volume and expressed as a percentage, is increasingly studied biomarker in clinical medicine [16,17]. An increase in RDW typically indicates the presence of anisocytosis, which can be a consequence of a delayed clearance of old RBCs, RBC underproduction, presence of a pro-inflammatory state, or reflect physiological conditions such as advancing age, pregnancy, and exercise [17,18,19,20,21]. Studies have reported the potential clinical utility of the RDW in specific disease states, e.g., chronic obstructive pulmonary disease (COPD) [22], immune disorders [23,24], cancer [25], surgical procedures [26], retinal artery occlusion [27], and COVID-19 [28,29,30]. In OSAS patients, a significant increase in the RDW has also been shown to be positively associated with increasing disease severity and negatively associated with oxygen saturation. The activation of pro-inflammatory factors as a consequence of intermittent hypoxia may explain the alterations of the RDW values in this group [31].

Therefore, we conducted a systematic review and meta-analysis to better investigate possible associations between the RDW and the presence and the severity of OSAS in order to determine the potential diagnostic role of this parameter in this patient group. 

## 2. Materials and Methods

### 2.1. Search Strategy, Study Selection, and Eligibility Criteria

A systematic review of published articles was performed using the following electronic databases: PubMed, Web of Science, Scopus, Embase, and Google Scholar. The search, from database inception to February 2023, focused on articles investigating the RDW in OSAS and non-OSAS subjects, and was conducted using the combinations of the following entries: “OSA”, “OSAS”, “obstructive sleep apnea syndrome”, “RDW”, “red cell distribution width”, “complete blood count”, “CBC”, “full blood count”, “FBC”, “anisocytosis”. 

After the removal of duplicate documents, titles and abstracts were screened by two independent investigators (BDL and AZ). Full texts of relevant manuscripts were independently reviewed by two investigators (BDL and AZ) before data extraction. Possible discrepancies were resolved by a third investigator (AAM). Prospective or retrospective observational studies were selected, whereas editorials, commentaries, basic research studies, or studies not written in English were excluded.

Studies of patients with OSAS on polysomnography (AHI ≥ 5) were included when their RDW was compared to that of non-OSAS subjects (5 < AHI). Studies of OSAS patients on treatment (e.g., continuous positive airway pressure and laryngectomy), pediatric patients (under 18 years), and patients with other sleep conditions (e.g., central sleep apnea) were excluded. The reference list from the selected manuscripts was also screened for additional studies.

### 2.2. Data Extraction

The following variables were independently collected by two investigators (BDL and AZ) from each article: year of publication, first author, study country, design of study (prospective or retrospective), sample size, age, sex, disease severity, AHI, RDW, body mass index (BMI), minimum and mean oxygen saturation (SpO2), oxygen desaturation index (ODI), smoking status, and history of hypertension (HPT), diabetes (DM), and cardiovascular diseases (CVD). Means and standard deviations were derived from medians and interquartile or actual ranges according to Wan et al. [32].

### 2.3. Quality Assessment and Certainty of Evidence

The Joanna Briggs Institute Critical Appraisal Checklist for analytical studies, consisting of eight items, was used to assess the risk of bias: a score of <4, 4, and ≥5 indicated, respectively, a high, moderate, and low risk of bias [33]. The evaluation of the certainty of evidence was performed using the Grades of Recommendation, Assessment, Development and Evaluation (GRADE) Working group system, based on the study design, the risk of bias, the presence of unexplained heterogeneity, the indirectness of evidence, the imprecision of results, the effect size, and the publication bias [34,35,36]. The Preferred Reporting Items for Systematic Reviews and Meta-Analysis (PRISMA) statement was followed [37]. The study protocol was registered in the International Prospective Register of Systematic Reviews (PROSPERO, CRD42023398047).

### 2.4. Statistical Analysis

Differences in RDW between OSAS and non-OSAS subjects were assessed using standardized mean differences (SMD), 95% confidence intervals (CIs), and *p* values (significance level < 0.05) in forest plots. Q statistic (significance level < 0.10) and the I^2^ statistic (I^2^ < 25%, no heterogeneity; 25% ≤ I^2^ < 50%, moderate heterogeneity; 50% ≤ I^2^ < 75%, large heterogeneity; I^2^ > 75%, extreme heterogeneity) were used to evaluate SMD heterogeneity and inconsistency across studies, respectively [38,39]. Fixed or random-effects models were used depending on the between-study heterogeneity (threshold at ≥50%). The influence of each study on the overall effect size was analyzed using a sensitivity analysis [40]. To investigate the presence of publication bias, Begg’s adjusted rank correlation test and Egger’s regression asymmetry test (*p* < 0.05) were used [41,42,43]. Univariate meta-regression analyses were executed to evaluate associations between effect size and age, gender, BMI, sample size, publication year, mean SpO_2_, min SpO_2_, ODI, smoking status, DM, HPT, and CVD. Statistical analyses were performed using Stata 14 (STATA Corp., 4905 Lakeway Dr, College Station, TX, USA). 

## 3. Results

### 3.1. Systematic Research and Study Characteristics

**Figure 1:**
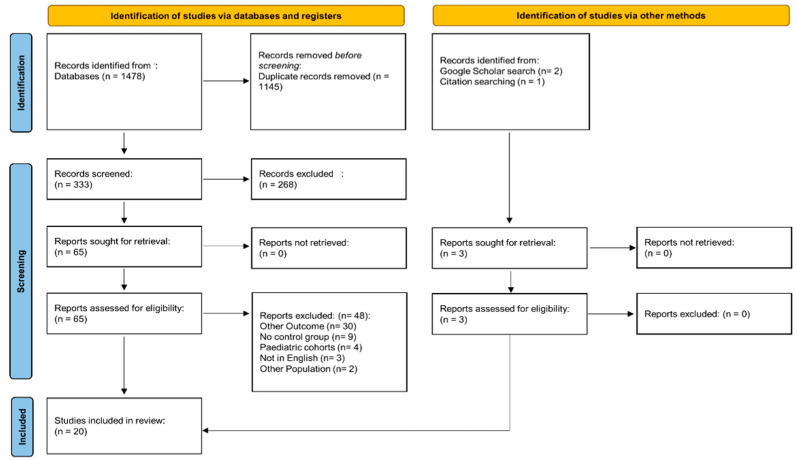
PRISMA 2020 flow diagram.

Initially, a total of 1478 articles were identified. Of them, 1145 were duplicates, and the remaining 333 were assessed through the screening of titles and abstracts. In total, 65 eligible articles were further appraised. Of these, 48 were removed because they did not report the studied endpoint (*n* = 30), did not include the control group (*n* = 9), investigated pediatric cohorts (*n* = 4) or different patient groups (*n* = 2), or were not written in English (*n* = 3), leaving 17 articles. Since three additional records were identified from Google Scholar and reference lists, a total of 20 manuscripts [14,44,45,46,47,48,49,50,51,52,53,54,55,56,57,58,59,60,61,62] were included in the final analysis (Figure 1). The included studies were published between 2012 [44] and 2022 [60,61,62]. The majority were conducted in Turkey (*n* = 15) [14,44,45,46,47,48,52,54,55,57,58,60,62], whereas the remaining (*n* = 5) were conducted in Spain [49], Egypt [50], Pakistan [53], China [56], and Romania [61]. Eight studies were retrospective [48,51,52,54,56,57,58,62] (Table 1).

The selected studies evaluated a total of 4528 OSAS (age 44.9 ± 11.1, 49.8% males) and 1335 non-OSAS (age 49.7 ± 10.8, 59.8% males) subjects, with an average RDW of 15.4 ± 1.5% and 14.8 ± 1.3%, respectively (Table 1). Comorbidities were present in 43.6% OSAS and 17.7% non-OSAS subjects (DM: 22.5% and 22.0%; HPT: 59.0% and 54.7%; CVD: 18.4% and 23.3%). In the OSAS and non-OSAS cohorts, the AHI was 37.2 ± 16.1 and 2.4 ± 1.3, the BMI was 31.7 ± 5.1 and 28.2 ± 4.1, the minimum SpO_2_ was 75.1 ± 9.6 and 87.7 ± 7.9, and the mean SpO_2_ was 88.6 ± 5.2 and 96.5 ± 16.4, respectively (Table 2).

### 3.2. Risk of Bias

The risk of bias was considered low in all studies (Table 3).

### 3.3. Results of Individual Studies and Syntheses

**Figure 2:**
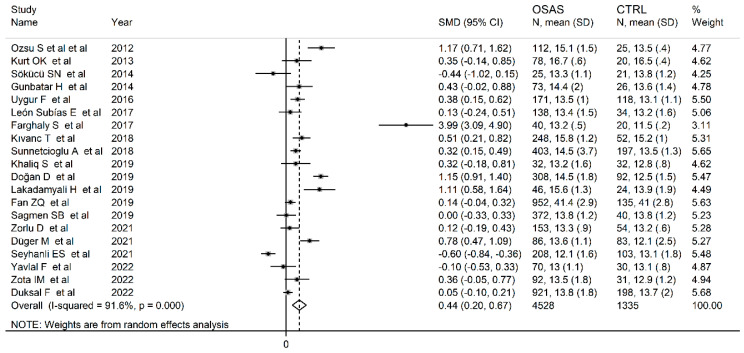
Forest plot of studies examining RDW values of OSAS patients and controls.

**Figure 3:**
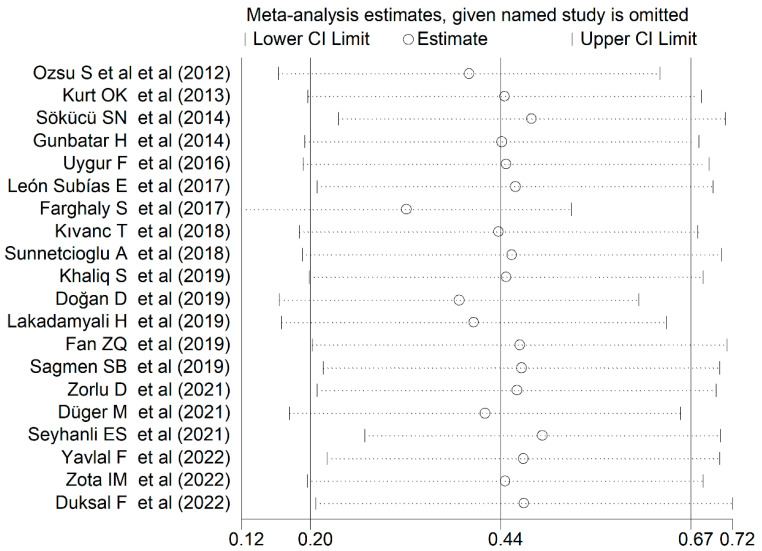
Sensitivity analysis of the association between RDW values and OSAS disease. For each study, the displayed effect size (hollow circles) corresponds to an overall effect size computed from a meta-analysis excluding that study.

**Figure 4:**
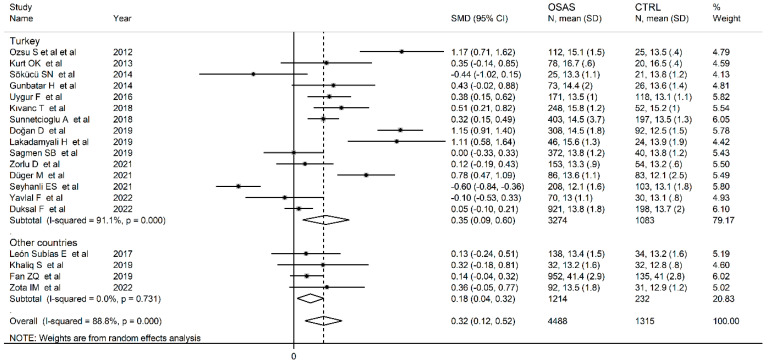
Forest plot of studies examining RDW values in OSA patients and controls according to the continent where the study was conducted.

The forest plot for RDW values in OSAS and non-OSAS subjects is described in Figure 2. Three studies [46,58,60] reported lower RDW values in OSAS subjects than non-OSAS controls (mean difference range, −0.10 to −0.60), with one reporting a statistically significant difference [60], whereas no between-group difference was reported in another study [57]. In the remaining 16 studies [14,44,45,47,48,49,50,52,53,54,55,56,58,61,62], OSAS patients had higher RDW values than non-OSAS subjects (mean difference range, 0.05 to 3.99), with a significant difference in eight [45,47,48,51,52,53,61]. Since a substantial heterogeneity between studies was observed (I^2^ = 91.6%, *p* < 0.001), random-effects models were used. Overall, pooled results showed that the RDW values were significantly higher in OSAS patients (SMD = 0.44, 95% CI 0.20 to 0.67; *p* < 0.001). The sensitivity analysis showed that the corresponding pooled SMDs were not substantially affected by sequentially removing individual studies (effect size range between 0.32 and 0.49, Figure 3). Nevertheless, the Funnel plot for bias detection analysis showed a distortive effect of one study [50] (Figure 4). Its removal attenuated the effect size (SMD 0.32, 95% CI 0.12 to 0.52; *p* = 0.002) but not the heterogeneity (I^2^ = 91.6%, *p* < 0.001).

### 3.4. Publication Bias

The *p*-values of the Begg and Egger tests were 0.62 and 0.41, respectively, highlighting no evidence of publication bias.

### 3.5. Meta-Regression and Sub-Group Analysis

**Figure 5:**
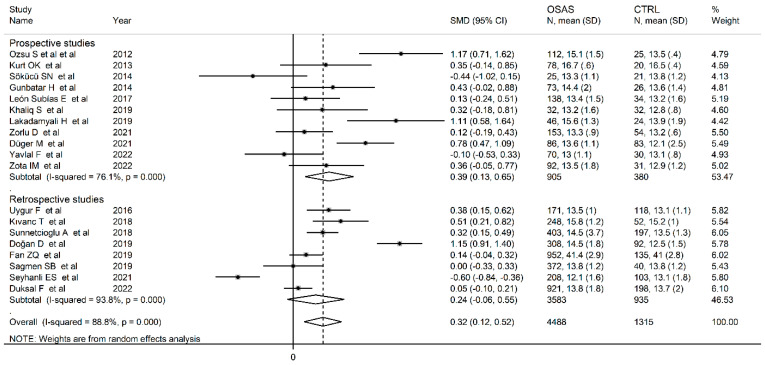
Forest plot of studies examining RDW values in OSA patients and controls according to study design.

Using univariate meta-regression analysis, no significant associations were observed between the effect size and age (t = −0.52, *p* = 0.61), gender (t = −0.37, *p* = 0.72), BMI (t = −0.45, *p* = 0.66), sample size (t = −0.71, *p* = 0.49), min SpO2 (t = −1.81, *p* = 0.11), publication year (t = −1.07, *p* = 0.30), ODI (t = −1.36, *p* = 0.25), smoking status (t = 0.03, *p* = 0.97), DM (t = −1.99, *p* = 0.09), HPT (t = −0.05, *p* = 0.96), and CVD (t = −0.39, *p* = 0.71). In contrast, the mean SpO_2_ was significantly correlated with effect size (t = −2.77, *p* = 0.02). Sub-group analysis did not reveal any significant between-group differences (*p* = 0.68) in RDW values from studies performed in Turkey (SMD = 0.35; 95% CI 0.09 to 0.60, *p* = 0.007; I^2^ = 91.1.0%, *p* < 0.001) and other countries (SMD = 0.18; 95% CI 0.04 to 0.32, *p* = 0.02; I^2^ = 0.0%, *p* = 0.73) (Figure 4). Interestingly, the between-study variance was virtually eliminated in the sub-group of studies conducted in other countries. A second sub-group analysis based on study design revealed no significant differences (*p* = 0.53) in RDW values between prospective (SMD = 0.39; 95% CI 0.13 to 0.65, *p* = 0.002; I^2^ = 76.1%, *p* < 0.001) and retrospective studies (SMD = 0.24; 95% CI −0.6 to 0.55, *p* = 0.12; I^2^ = 93.8%, *p* < 0.001) (Figure 5), even though the effect size in retrospective studies was not statistically significant.

**Figure 6:**
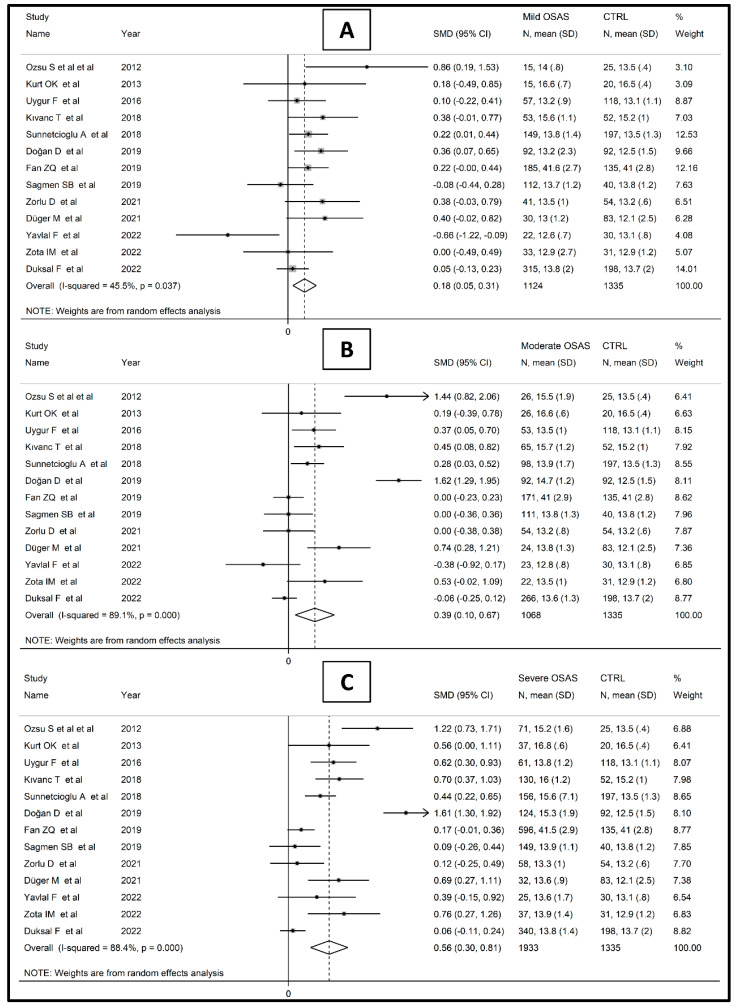
Forest plot of studies examining RDW values of mild (**A**), moderate (**B**), and severe OSAS patients (**C**) vs. controls.

**Figure 7:**
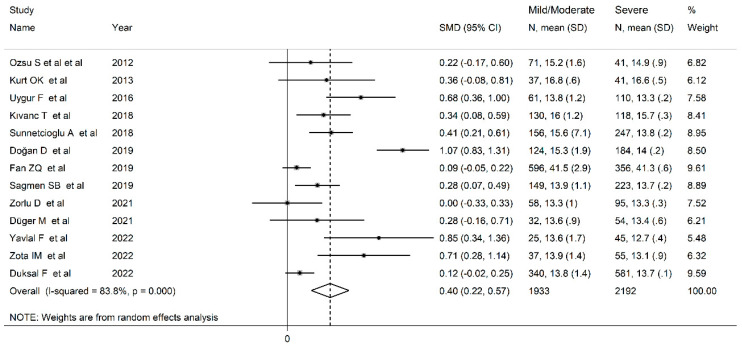
Forest plot of studies examining RDW values of mild/moderate vs. severe OSAS patients.

Finally, 13 studies [14,44,45,48,51,52,54,56,57,58,60,61,62] also reported RDW values based on disease severity. RDW values in OSAS subjects were significantly higher in comparison to non-OSAS controls, in a progressive manner, from mild (SMD = 0.18; 95% CI 0.05 to 0.314, *p* = 0.006; I^2^ = 45.5%, *p* = 0.037, Figure 6A) to moderate (SMD = 0.39; 95% CI 0.10 to 0.67, *p* = 0.009; I^2^ = 89.1%, *p* < 0.001, Figure 6B) and to severe disease (SMD = 0.56; 95% CI 0.30 to 0.81, *p* < 0.001; I^2^ = 88.4%, *p* < 0.001, Figure 6C). RDW values were also significantly higher in subjects with the severe form of the disease than patients with mild/moderate OSAS (SMD = 0.40, 95% CI 0.22 to 0.57, *p* < 0.001; I^2^ = 83.8%, *p* < 0.001, Figure 7).

### 3.6. Certainty of Evidence

The initial level of certainty was regarded as low because of the cross-sectional nature of the studies (rating 2, ⊕⊕⊝⊝). Considering the low risk of bias in all studies (no rating change), the substantial but partially explainable heterogeneity (no rating change), the lack of indirectness (no rating change), the relatively low imprecision (confidence intervals not crossing the threshold, no rating change), the relatively small effect size (SMD = 0.44, no rating change), and the publication bias absence (no rating change), the overall level of certainty remained low (rating 2, ⊕⊕⊝⊝).

## 4. Discussion

Although OSAS can occur in both genders at any age, it has a relatively higher prevalence in middle-aged males [63,64,65]. Typically, OSAS coexists with several comorbidities, particularly DM, HPT, and CVD, and it carries a 2–3-fold increased risk of progressing to cardiovascular end-organ dysfunction [66]. In this regard, relatively high circulating concentrations of markers of vascular pathology, renal dysfunction, and inflammation, e.g., 1-methylhistidine, symmetric (SDMA) and asymmetric (ADMA) dimethylarginine, have been reported in aging cohorts [67].

Nevertheless, the investigation of the role of systemic inflammation and vasculo-renal dysfunction has failed to identify circulating biomarkers for early OSAS diagnosis and progression [66].

In a previous meta-analysis [15], Wu M et al. identified an association between OSAS and several hematologic parameters, specifically WBC, neutrophil-to-lymphocyte ratio (NLR), MPV, PDW, platelet-to-lymphocyte ratio (PLR), hematocrit (HCT), and RDW. The latter association was observed after extracting information from five studies. Additionally, higher RDW values have been shown to be associated with intermittent hypoxic events, oxidative stress, endothelial dysfunction, upregulation of pro-inflammatory transcription factors, and increase in inflammatory cells in OSAS patients [31,68]. In this context, a study reported the activation of the ERK and NF-κB inflammatory pathways using an in vitro model of desaturation and reoxygenation cycles mimicking intermittent hypoxic cycles. This activation favored the overexpression of ICAM-1 and CCL2, pro-inflammatory adhesion, and signaling molecules, respectively [69].

In light of this, we investigated the potential diagnostic role of the RDW as this easily accessible hematological parameter is associated with oxidative stress and inflammation [70]. The RDW has been shown to strongly predict all-cause mortality in aging populations [71,72] in the context of cardiovascular and respiratory disease states. However, the pathophysiological mechanism underlying this association is still unclear. In a previous report, the RDW was associated with an increased response to hypoxemia, with the formation of larger erythrocytes [73]. These could alter both blood flow patterns and the interaction with other blood elements and the endothelium, facilitating the development of atherosclerosis [74]. Moreover, the RDW has been associated with an increase in AHI [70] and alterations in ODI and saturation indexes [44,45,46,47,48,52,55,56,57,60,70].

In our systematic review and meta-analysis, the RDW was higher in OSAS subjects, and progressively increased with disease severity. 

Moreover, the overall SMD values were not significantly altered in the sensitivity analysis, in spite of the presence of substantial heterogeneity. Only removing the study published by Farghaly S et al. [50] mildly attenuated the effect size because of a distortive effect, probably due to the relatively low dispersion. Furthermore, no publication bias was identified. The univariate meta-regression analysis showed no significant associations between the effect size and study and patient characteristics, barring mean SpO_2_.

It is uncertain whether continuous positive airway pressure (CPAP) therapy can reduce the RDW while ameliorating OSAS severity [49,55,61,75]. Unfortunately, our search strategy did not identify a sufficient number of studies for analysis, which justifies the conduct of further studies to clarify this issue. Future studies should also investigate the sleep apnea specific hypoxic burden (SASHB) [76], an oxygenation index extensively used to study cardiovascular outcomes in OSAS subjects, its associations with the RDW, and the role of both indices in predicting hearth failure.

Finally, although no significant differences were observed in subgroup analysis, specific study geographical locations represented a source of heterogeneity. Additionally, the inclusion of eight retrospective studies could have added a selection bias in our analysis. These aspects, together with the observed low risk of bias, low imprecision, and absence of publication bias maintained the initial level of certainty. However, one of the main limitations of our study was the great difference in sample size when comparing OSAS and non-OSAS subjects. Although we collected RDW measurements with relatively low dispersion from a high number of subjects, it remains possible that increasing the sample size of the control groups would reduce the observed differences in RDW. Furthermore, it was not possible to assess how the RDW changes in OSAS subjects when compared to a healthy group, as the majority of the studies included subjects with comorbidities.

## 5. Conclusions

This systematic review and meta-analysis demonstrated that RDW values are significantly increased in OSAS patients compared to non-OSAS subjects, and that the increase is positively related to disease severity. These findings, together with the reduction in the mean SpO_2_, highlight the presence of an underlying pathological mechanism probably linked to the reduction of oxygenation and inflammation. Nevertheless, given the high heterogeneity estimated in the included studies, future research aiming at confirming the relation between RDW and OSAS should be conducted employing standardized methods and diagnostic criteria. 

## Figures and Tables

**Table 1:** Summary of study characteristics, demographics, and detected RDW. All RDW values are expressed as coefficient of variation (RDW-CV) except for the study from Fan ZQ. * RDW expressed as standard deviation (RDW-SD); P: prospective; R: retrospective; SD: standard deviation.

First Author	Year	Country	Study Design	Non-OSA				OSA			
				Sample Size	Age (Mean ± SD)	Gender (M/F)	RDW (Mean ± SD)	Sample Size	Age (Mean ± SD)	Gender (M/F)	RDW (Mean ± SD)
Ozsu S et al. [44]	2012	Turkey	P	25	47.5 ± 7.3	07/18	13.5 ± 0.43	112	51.1 ± 7.9	78/34	15.08 ± 1.53
Kurt OK et al. [45]	2013	Turkey	P	20	46.3 ± 13.1	11/9	16.5 ± 0.4	78	55.5 ± 11	51/27	16.69 ± 0.62
Sökücü SN et al. [46]	2014	Turkey	P	21	40.8 ± 11.6	21/0	13.84 ± 1.19	25	47.4 ± 11.7	25/0	13.34 ± 1.09
Gunbatar H et al. [47]	2014	Turkey	P	26	41.3 ± 11	-	13.6 ± 1.4	73	50.8 ± 11.7	45/28	14.4 ± 2
Uygur F et al. [48]	2016	Turkey	R	118	50.3 ± 11.7	61/57	13.1 ± 1.1	171	53.4 ± 11.9	105/66	13.51 ± 1.04
León Subías E et al. [49]	2017	Spain	P	34	40.5 ± 9.8	19/15	13.15 ± 1.6	138	45.6 ± 9.2	110/28	13.4 ± 1.48
Farghaly S et al. [50]	2017	Egypt	P	20	49.8 ± 10.28	8/12	11.5 ± 0.18	40	48.9 ± 9.1	13/27	13.15 ± 0.47
Kıvanc T et al. [51]	2018	Turkey	R	52	41 ± 12	35/17	15.2 ± 1.02	248	49 ± 10.8	192/56	15.84 ± 1.17
Sunnetcioglu A et al. [52]	2018	Turkey	R	197	40 ± 11.5	152/45	13.5 ± 1.3	403	47.1 ± 11.4	304/99	14.52 ± 3.68
Khaliq S et al. [53]	2019	Pakistan	P	32		-	12.76 ± 0.82	32	-	-	13.22 ± 1.58
Doğan D et al. [54]	2019	Turkey	R	92	34.8 ± 12.5	-	12.5 ± 1.5	308	39.1 ± 12.8	-	14.49 ± 1.81
Lakadamyali H et al. [55]	2019	Turkey	P	24	47.9 ± 13.2	16/08	13.9 ± 1.9	46	52.2 ± 12.7	38/8	15.6 ± 1.3
Fan ZQ et al. * [56]	2019	China	R	135	46.3 ± 12	135/0	40.96 ± 2.79	952	44.2 ± 11	952/0	41.43 ± 2.86
Sagmen SB et al. [57]	2019	Turkey	R	40	49.2 ± 11	-	13.8 ± 1.2	372	48.5 ± 12	-	13.8 ± 1.2
Zorlu D et al. [14]	2021	Turkey	P	54	53.1 ± 11.2	26/28	13.2 ± 0.59	153	54.4 ± 12.1	95/58	13.33 ± 0.92
Düger M et al. [58]	2021	Turkey	P	83	42.8 ± 14	48/35	12.08 ± 2.5	86	45.1 ± 3.2	54/32	13.6 ± 1.1
Seyhanli ES et al. [59]	2021	Turkey	R	103	46.9 ± 9.2	-	13.1 ± 1.84	208	54.4 ± 10.9	-	12.07 ± 1.63
Yavlal F et al. [60]	2022	Turkey	P	30	42.1 ± 2.4	9/21	13.05 ± 0.8	70	51.5 ± 12.6	51/19	13 ± 1.09
Zota IM et al. [61]	2022	Romania	P	31	49.6 ± 14	16/15	12.94 ± 1.24	92	56.7 ± 11.5	62/30	13.45 ± 1.77
Duksal F et al. [62]	2022	Turkey	R	198	42.3 ± 12.1	101/97	13.73 ± 1.98	921	50 ± 11.4	534/387	13.75 ± 1.8
Total				1335	44.9 ± 11	665/377	14.8 ± 1.3	4528	49.7 ± 10.8	2709/899	15.4 ± 1.5

**Table 2:** Summary of patient clinical characteristics and comorbidities. DM: diabetes mellitus; HPT: hypertension; CVD: cardiovascular disease; BMI: body mass index; AHI: apnea-hypopnea index; minSpO_2_: minimal oxygen saturation; meanSpO_2_: average oxygen saturation.

First Author	Non-OSA									OSA								
	DM	HPT	CVD	BMI	AHI	minSpO_2_	meanSpO_2_	ODI	Smokers	DM	HPT	CVD	BMI	AHI	minSpO_2_	meanSpO_2_	ODI	Smokers
Ozsu S et al. [44]	3 (12)	9 (36)	2 (8)	31.5 ± 2.7	2.5 ± 1.4	79.1 ± 26	94.8 ± 1.5	2.3	13 (52)	15 (60)	80 (320)	20 (80)	35.9 ± 6.5	44.6 ± 15.3	70.1 ± 9.9	84.7 ± 5.4	39.0	65 (260)
Kurt OK et al. [45]	-	5 (25)	0 (0)	29.4 ± 4.9	-	89.4 ± 3	-	-	5 (25)	18 (90)	33 (165)	7 (35)	31.8 ± 4.9	-	76.1 ± 9.1	-	-	16 (80)
Sökücü SN et al. [46]	-	5 (23.8)	-	28.7 ± 6.1	3.4 ± 1.19	91.4 ± 2	96.8 ± 1.1	3.0	14 (66.7)	-	9 (42.9)	-	33.1 ± 4.4	59.3 ± 19	75 ± 10.3	94.6 ± 1.4	54.6	17 (81)
Gunbatar H et al. [47]	20 (76.9)	-	-	27.3 ± 4.5	2.6 ± 1.4	-	-	-	-	-	-	-	32.9 ± 5	32.2 ± 26.8	73.6 ± 10.3	85.9 ± 11.6	-	-
Uygur F et al. [48]	-	25 (21.2)	24 (20.3)	29.4 ± 7.8	2.2 ± 1.3	-	92.4 ± 3.5	1.8	43 (36.4)	34 (28.8)	55 (46.6)	65 (55.1)	31.5 ± 6.9	31.1 ± 8.5	-	80.2 ± 8.5	24.1	63 (53.4)
León Subías E et al. [49]	-	-	-	25.9 ± 3.3	-	-	-	-	-	-	-	-	31.1 ± 4.1	-	-	-	-	-
Farghaly S et al. [50]	2 (10)	-	-	27.5 ± 1.3	-	-	-	-	-	-	-	-	28.8 ± 2.9	38.15	-	-	56.2	-
Kıvanc T et al. [51]	12 (23.1)	11 (21.2)	8 (15.4)	29 ± 4.3	2.2 ± 1.5	89 ± 4.4	110 ± 117	2.1	29 (55.8)	52 (100)	102 (196.2)	43 (82.7)	32.2 ± 5.2	39.6 ± 14.4	77.4 ± 7.6	88.33 ± 4.8	37.4	116 (223.1)
Sunnetcioglu A et al. [52]	-	19 (9.6)	12 (6.1)	28.3 ± 4.7	2.6 ± 1.4	85.5 ± 8.7	-	-	26 (13.2)	58 (29.4)	137 (69.5)	71 (36)	31.7 ± 6.3	30.9 ± 11.4	76.1 ± 9.2	-	-	94 (47.7)
Khaliq S et al. [53]	-	-	-	-	-	-	-	-	-	-	-	-	-	-	-	-	-	-
Doğan D et al. [54]	-	-	-	26.2 ± 3	-	-	-	-	-	-	-	-	28.7 ± 3.7	-	-	-	-	-
Lakadamyali H et al. [55]	9 (37.5)	-	-	28.4 ± 5.4	2.5 ± 0.8	91.1 ± 2.1	95 ± 1	3.8	12 (50)	-	-	-	36.6 ± 6.2	27.4 ± 14	72 ± 12.9	84.5 ± 5.7	8.3	9 (37.5)
Fan ZQ et al. [56]	-	23 (17)	-	24 ± 3.32	2.1 ± 1.4	88.1 ± 5.3	95.9 ± 1.5	-	-	140 (103.7)	299 (221.5)	-	27.9 ± 6.1	43 ± 12.7	72.2 ± 9.3	92.4 ± 3.4	-	-
Sagmen SB et al. [57]	-	-	-	29.9 ± 4.9	2.3 ± 1.4	90.4 ± 2.2	95 ± 1.7	-	-	-	-	-	31.1 ± 5.4	29.4 ± 25.8	82.3 ± 8.3	92.9 ± 4	-	-
Zorlu D et al. [14]	-	-	-	-	-	-	-	-	-	-	-	-	-	-	-	-	-	-
Düger M et al. [58]	-	-	-	30.9 ± 2.3	-	-	-	-	18 (21.7)	-	-	-	32.3 ± 5.9	-	-	-	-	14 (16.9)
Seyhanli ES et al. [59]	-	-	-	24.3 ± 1	-	-	-	-	-	100 (97.1)	58 (56.3)	24 (23.3)	29.7 ± 2.5	-	-	-	-	-
Yavlal F et al. [60]	6 (20)	-	-	26.5 ± 5.7	1.6 ± 1.2	85.1 ± 17.5	96.1 ± 17.8	-	-	-	-	-	30.3 ± 4.7	33.7 ± 13.2	76.5 ± 9	92.6 ± 3	-	-
Zota IM et al. [61]	-	-	-	32.1 ± 5.2	-	-	-	-	5 (16.1)	28 (90.3)	-	-	33.6 ± 5.7	-	-	-	-	11 (35.5)
Duksal F et al. [62]	-	32 (16.2)	9 (4.5)	-	-	-	92.8 ± 2.1	3.9	-	-	392 (198)	134 (67.7)	-	-	-	89.7 ± 4.6	29.8	-
Total	52 (3.9)	129 (9.7)	55 (4.1)	28.2 ± 4.1	2.4 ± 1.3	87.7 ± 7.9	96.5 ± 16.4	2.8 ± 0.9	165 (12.4)	445 (33.3)	1165 (87.3)	364 (27.3)	31.7 ± 5.1	37.2 ± 16.1	75.1 ± 9.6	88.6 ± 5.2	35.6 ± 16.9	405 (30.3)

**Table 3:** The Joanna Briggs Institute critical appraisal checklist.

Study	Were the Criteria for Inclusion Clearly Defined?	Were the Subjects and the Setting Described in Detail?	Was the Exposure Measured in a Valid and Reliable Way?	Were Objective, Standard Criteria Used for Measurement of the Condition?	Were Confounding Factors Identified?	Were Strategies to Deal with Confounding Factors Stated?	Were the Outcomes Measured in a Valid and Reliable Way?	Was Appropriate Statistical Analysis Used?	Risk of Bias
Ozsu S et al., 2012 [44]	Yes	Yes	Yes	Yes	Yes	Yes	Yes	Yes	Low
Kurt OK et al., 2013 [45]	Yes	Yes	Yes	Yes	No	No	Yes	No	Low
Sökücü SN et al., 2014 [46]	Yes	Yes	Yes	Yes	No	No	Yes	No	Low
Gunbatar H et al., 2014 [47]	Yes	Yes	Yes	Yes	No	No	Yes	No	Low
Uygur F et al., 2016 [48]	Yes	Yes	Yes	Yes	No	No	Yes	No	Low
León Subías E et al., 2017 [48]	Yes	Yes	Yes	Yes	Yes	Yes	Yes	Yes	Low
Farghaly S et al., 2017 [49]	Yes	Yes	Yes	Yes	No	No	Yes	No	Low
Kıvanc T et al., 2018 [50]	Yes	Yes	Yes	Yes	No	No	Yes	No	Low
Sunnetcioglu A et al., 2018 [51]	Yes	Yes	Yes	Yes	No	No	Yes	No	Low
Khaliq S et al., 2019 [52]	Yes	Yes	Yes	Yes	No	No	Yes	No	Low
Doğan D et al., 2019 [54]	Yes	Yes	Yes	Yes	No	No	Yes	No	Low
Lakadamyali H et al., 2019 [55]	Yes	Yes	Yes	Yes	Yes	Yes	Yes	Yes	Low
Fan ZQ et al., 2019 [56]	Yes	Yes	Yes	Yes	Yes	Yes	Yes	Yes	Low
Sagmen SB et al., 2019 [57]	Yes	Yes	Yes	Yes	No	No	Yes	No	Low
Zorlu D et al., 2021 [14]	Yes	Yes	Yes	Yes	No	No	Yes	No	Low
Düger M et al., 2021 [58]	Yes	Yes	Yes	Yes	Yes	Yes	Yes	Yes	Low
Seyhanli ES et al., 2021 [59]	Yes	Yes	Yes	Yes	No	No	Yes	No	Low
Yavlal F et al., 2022 [60]	Yes	Yes	Yes	Yes	No	No	Yes	No	Low
Zota IM et al., 2022 [61]	Yes	Yes	Yes	Yes	Yes	Yes	Yes	Yes	Low
Duksal F et al., 2022 [62]	Yes	Yes	Yes	Yes	No	No	Yes	No	Low

## Data Availability

All data relevant to the study are included in the article.
